# NGF, BDNF, and NO in Myopic Subjects: Relationships Between Aqueous Levels and Lens Epithelial Cells’ Activation

**DOI:** 10.3390/ijms26136350

**Published:** 2025-07-01

**Authors:** Maria De Piano, Andrea Cacciamani, Fabio Scarinci, Rosanna Squitti, Pamela Cosimi, Marisa Bruno, Guido Ripandelli, Paola Palanza, Alessandra Micera

**Affiliations:** 1 Research and Development Laboratory for Biochemical, Molecular and Cellular Applications in Ophthalmological Science, IRCCS-Fondazione Bietti, 00184 Rome, Italy; maria.depiano@fondazionebietti.it; 2 Surgical Retina Research Unit, IRCCS-Fondazione Bietti, 00184 Rome, Italy; andrea.cacciamani@gmail.com (A.C.); fabioscarinci@gmail.com (F.S.); pcosimi@gmail.com (P.C.); marisa.bruno@fondazionebietti.it (M.B.); guido.ripandelli@fondazionebietti.it (G.R.); 3 Department of Theoretical and Applied Sciences, eCampus University, Viale Massenzio Masia, 26, 22100 Novedrate, Italy; rosanna.squitti@afar.it; 4 Department of Laboratory Science, Research and Development Division, Ospedale Isola Tiberina–Gemelli Isola, 00186 Rome, Italy; 5 Department of Medicine and Surgery, University of Parma, 43126 Parma, Italy; paola.palanza@unipr.it

**Keywords:** myopia, LEC, NGF, BDNF, NO, neurodegeneration, stressful effectors

## Abstract

Several soluble mediators are activated during myogenesis and progression, and severe neurodegeneration, with related biomarkers, characterizes high myopia-related retinal atrophy. Targets of oxidative stress, epigenetics and neurogenic inflammation have been reported in the prospecting of some bioindicators to mirror retinal insults occurring in high myopia. The aim of the present study was to assess the expression of a few selected biomarkers belonging to the neurotrophin (NGF and BDNF), oxidative (NO, KEAP1/NRF2), and epigenetic (DNMT3 and HD1) pathways. Sixty-five (65; 76.25 ± 9.40 years) specimens—aqueous, anterior capsule (AC), and lens epithelial cells (LEC)—were collected at the time of cataract surgery and used for ELISA (aqueous) and transcripts analysis (AC/LEC). Biosamples were grouped as emmetrope (23; 81.00 ± 6.70 years); myopia (24; 75.96 ± 7.30); and high (pathological) myopia (18; 70.56 ± 11.68 years), depending on axial length (AL) and refractive error (RE). Comparisons and correlations were carried out between myopic and high-myopic subgroups. NGF and BDNF were lowered in myopic samples; *NGF* and *BDNF* transcripts were differentially expressed in LEC, and their expression correlated positively with *NGF* and negatively with *BDNF*, with the expression of the *αSMA* phenotype. *NGF* and *BDNF* correlated negatively with NO and nitrites. Oxidative stress (*iNOS*/*NOX1*/*NOX4* and *KEAP1*/*NRF2*) and epigenetic (*DNMTα3*/*HD1*) transcripts were upregulated in myopic LEC, compared with emmetropic ones. Herein, we prospect the contribution of NGF and BDNF in both neuroinflammation and neuroprotection occurring in this chronic disease.

## 1. Introduction

Myopia is a very common refractive error (22.9% myopia and 2.7% high myopia of the world population in 2000) that leads to blurred vision of images from a distance [1]. Myopia strategies include specific types of multifocal soft contact lenses, new design spectacle lenses, and atropine, which all slow myopia progression to some degree, while correction by single vision spectacles, reshaping of the cornea by laser, or crystalline lens implants, are all solutions that do not inhibit myopia progression [2]. Unfortunately, high myopia increases the probability of developing severe ocular changes such as cataracts, high intraocular pressure, and retinal detachment [3]. Pathological myopia refers to myopia susceptible to developing choroidal neovascularization (mCNV): in those cases, the stretching and thinning of the retinal structure, and ultimately the photoreceptors’ loss, occur, and for this reason, the complex network of the retina is monitored to detect eventual retinal degeneration and severe retinal neurodegeneration [2,3,4]. Today the high-resolution imaging provided by optical coherence tomography (OCT) allows us to assess myopic retinal changes, except for in cases with “opacities”, such as cataracts or vitreous hemorrhage, which can interfere with the imaging process [4,5]. Several studies reported the association of myopia with different growth factors, a few neuromediators and inflammatory mediators, all involved in the pathophysiological processes occurring particularly in high myopia having insulted retina [6]. Growth factor (TGF)-β, fibroblast growth factor (FGF), and insulin-like growth factor (IGF) were found to regulate scleral thickness influencing both development and severity of myopia [6]. The neurotrophins nerve growth factor (NGF) and brain-derived neurotrophic factor (BDNF) are known to increase upon eye inflammation, promote repair of optic nerve injury, allow corneal tissue and tear film remodeling while retaining functional activity, and finally play a crucial role in the homeostasis of the underneath retina, as reported in both experimental models and ex-vivo human tissues [7,8]. NGF and BDNF are pleiotropic factors that work as critical regulators of neural development and plasticity and synaptic function, implying a direct role in the regulation of proliferation, growth, differentiation, survival, and death of different neuronal and non-neuronal cells [9,10]. At the cellular level, NGF and BDNF regulate cell-to-cell interactions, drive wound healing repair (crush nerves), and contribute actively to tissue repair and ECM remodeling [10]. Although released upon injury and inflammation, NGF and BDNF are unable to trigger inflammation by themselves [10]. NGF and BDNF participate in several acute and chronic inflammatory and autoimmune scenarios, including multiple sclerosis (MS), Alzheimer’s disease (AD), also known as autoimmune diseases or as novel conceptualization, such as “type 3 diabetes”, working as biomarkers for vasculogenesis and angiogenesis, metabolite regulation (glucose, lipid, and antioxidant), and energy metabolism in several systems including the visual one [10,11,12,13]. Both NGF and BDNF have been associated with cataractogenesis, and particularly the expression of NGF was found to be impaired in UV light-exposed cells and experimental models [14,15,16].

NGF and BDNF signals by two specific and independent receptors, respectively, the high-affinity tyrosine kinase receptors trkA^NGFR^ (for NGF) and trkB^BDNFR^ (for BDNF), and both neurotrophins share the pan-neurotrophin low-affinity nonselective transmembrane glycoprotein receptor p75^NTR^ [17,18]. Upon specific receptor binding, a series of downstream signaling cascades occur (from MAPK, PLCγ/IP3, and PI3K/Akt to the ERC and cJun/JNK pathways), allowing the activation of several cell-to-tissue responses that focus on the modulation in gene expression and ion channel activity with the final regulation of neuronal and non-neuronal cell activities [17,18,19,20]. On the other side, NGF and BDNF have been associated with oxidative stress and the metabolism of nitric oxide (NO), a mediator crucially released at the beginning of inflammation and playing a relevant role in many intricate cellular routes alongside neurodegenerative events, as observed in the human retina [21].

Scleral hypoxia and nitric oxide synthetase (iNOS) expression, followed by nitric oxide (NO) release, were associated with the progression of myopia, as triggering myofibroblast trans-differentiation, collagen formation, and thinning of the sclera, typical features of myopia, suggest some regulatory effects on scleral ECM [22]. NO is the product of the action of NO synthase (iNOS), an oxidative enzyme induced upon immunological or inflammatory activators that is able to generate NO in large amounts and in a long-lasting fashion [22,23]. Finally, the nitrite reductase-induced NO can promote tissue vasodilation, helping the survival of cells by increasing blood flow even under hypoxic conditions [24,25,26]. Under oxidative stress conditions, excessive ROS production causes the NRF2/KEAP1 complex disaggregation and the migration of the phosphorylated form of NRF2 inside the nucleus, and that complexing with small Maf family members and binding to AREs ends with the transcription of antioxidant enzymes [27]. NRF2 (signal transcription factor) is mostly located in cytosol, as it is physiologically bound to its specific inhibitor KEAP1 [27]. The activated cytoplasmatic NRF2 disaggregates from KEAP1 and the phosphor-NRF2 protein moves to the nucleus to bind antioxidant response elements (AREs), mediating the transcription of related genes and synthesis of the final antioxidant products [27].

Several risk factors have been hypothesized from biological (growth factors and signal pathways) to genetic (genetic locus linkage) and epigenetic (environmental factors) factors [28]. Gene linkage and genome-wide association (GWA) studies were carried out to identify the molecular targets of disease-causing genes and drug development in myopia [28]. These studies (pubmed2ensembl–GenCLiP3 database, GO biological process—Reactome pathway enrichment analyses, protein–protein interaction network, and algorithm analysis) regarding the biomarkers of developing myopia and progressive pathological myopia were of great interest, as there was a lack of an effective mCNV animal model [29]. From these studies, it appeared that the sclera, RPE, choroid, and retina can be influenced by growth factors in the case of genetic and epigenetic predisposition to myopia [28,29]. Recent studies identified 55 potential genes related to choroid, neovascularization, and myopia [29]. The algorithm-based analysis selected 14 genes, comprising VEGFA, IL6, FGF2, MMP9, IL10, TNF, MMP2, HGF, MMP3, IGF1, CCL2, CTNNB1, EDN1, BDNF, and NGF myopia targets with diagnostic and therapeutic purposes [28,29]. Further gene enrichment analysis discovered 11 biological processes and seven signal pathways [28]. In parallel, a single nucleotide polymorphism (SNP) analysis identified the expression gene pattern of FGF2, BDNF, COL2A1, COL18A1, and PAX6 [28]. Several biomarkers of retinal inflammation have been investigated in mCNV, such as IL6, FGF2, MMP9, IL10, TNF, MMP2, HGF, MMP3, IGF1, CCL2, CTNNB1, and EDN1 [29,30,31].

We recently demonstrated that changes in aqueous and vitreous protein signature can be detectable in different human ocular disorders, highlighting also the possibility of mirroring the status of the retina under pathological conditions, including myopia with cataract [14]. Since NGF and BDNF were included in the list of genes related to choroid, neovascularization, and myopia, and since these neurotrophins exert crucial neuroprotective efforts on insulted retina, the aim of the present study was to characterize their levels in myopic aqueous and the related protein/receptor expression by lens epithelial cells (LEC) from subjects who underwent cataract surgery. This biochemical and molecular approach was implemented by studies of the correlation between these neurotrophins and a few oxidative stress mediators, nitric effectors, and epigenetic targets quantified in parallel in ocular fluids and LEC.

## 2. Results

According to the aim of the study, biosamples were categorized into the following subgroups depending on axial length (AL) and refractive error (RE): emmetropia was defined as SE between −0.50 D and + 0.50 D; myopia as SE ≤ −0.50 D, and AL between 24 and 26 mm; and high myopia as SE ≤ −6.00 D and AL ≥ 26 mm. The characteristics of this study population comprising emmetropia, myopia, and high myopia are summarized in Table 1. In our study population, high myopia is clinically defined as an axial length of the eye equal to or greater than 26.0 mm or a refractive error of −6.00 diopters or more, often associated with degenerative changes in the retina and other ocular structures.

### 2.1. NGF and BDNF Proteins Humor Aqueous Decreased with Myopia Severity

**Figure 1:**
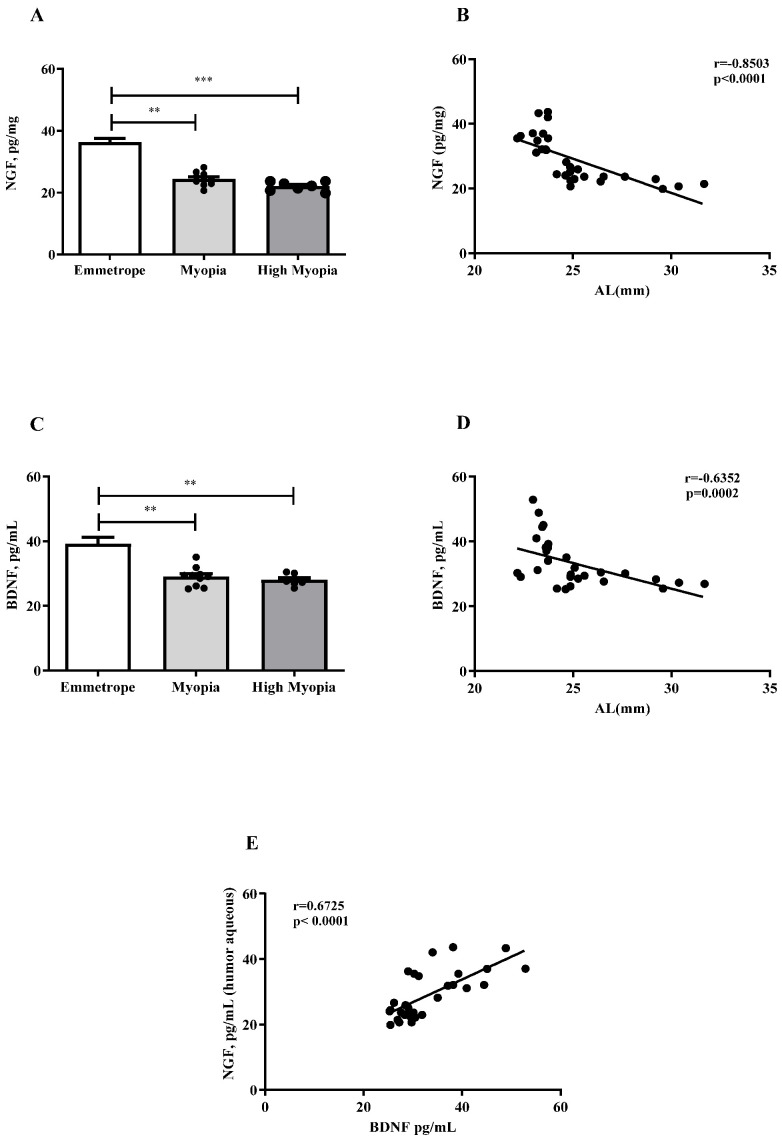
NGF and BDNF levels in aqueous from myopic patients. ELISA assay detected decreasing protein levels of NGF (**A**) and BDNF (**C**) in correlation with increasing axial length (**B**,**D**). The Spearman rho test showed that NGF correlated positively with BDNF (**E**). Significant levels are shown in the panels (** *p* ≤ 0.005; *** *p* ≤ 0.0005), as calculated using the Kruskal–Wallis test followed by a Dunn’s post-hoc test (mean ± SEM). Correlation data (coefficient and *p* value) are shown in the plot.

As shown in Figure 1, NGF (Figure 1A) and BDNF (Figure 1C) protein levels were significantly decreased in humor aqueous collected from patients with myopia (respectively, *p* < 0.005 and *p* < 0.005) and high myopia (respectively, *p* < 0.0005 and *p* < 0.005) with respect to emmetrope, while no difference was detected between myopia and emmetrope (*p* > 0.05). The Spearman rho test analysis revealed an inverse correlation for NGF/AL (r = −0.8503, *p* < 0.0001; Figure 1B) and BDNF/AL (r = −0.6352, *p* = 0.0002; Figure 1D), and a direct correlation between NGF and BDNF (r = 0.6725, *p* < 0.0001; Figure 1E), as expected.

### 2.2. NGF and BDNF Transcripts Were Differentially Expressed in Anterior Capsule (LEC)

**Figure 2:**
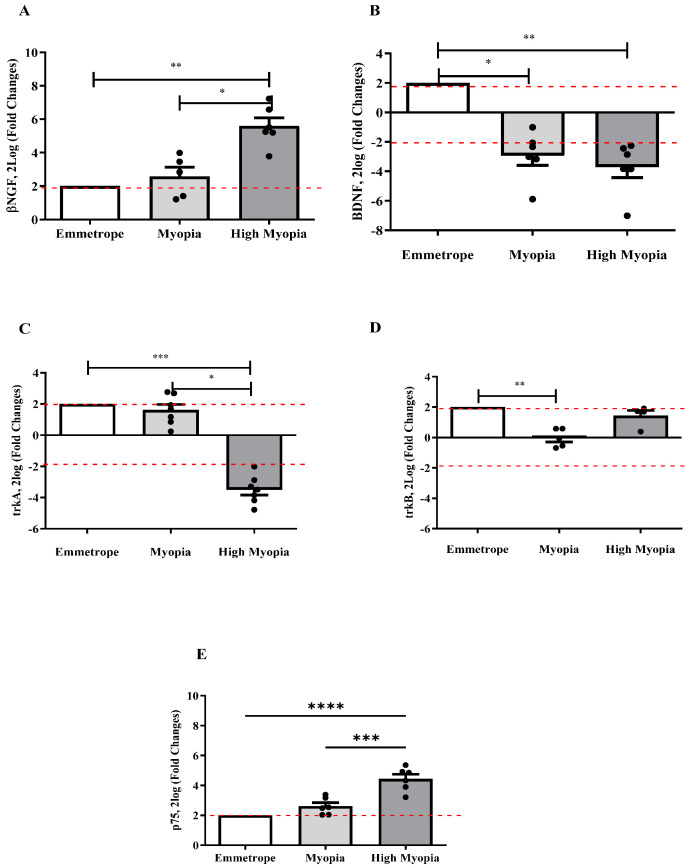
Transcript expression of NGF and BDNF pathways in LEC. Bar graph showing the transcript expression specific for NGF and BDNF pathways: (**A**) *βNGF*mRNA upregulation and (**B**) *BDNF*mRNA downregulation as functions of myopia severity. A consistent transcript deregulation was observed for *trkA^NGFR^* (**C**) as a function of disease severity, while *trkB^BDNFR^* deregulation (**D**) was observed specifically for myopia. The pan-neurotrophin *p75^NTR^* mRNA increased depending on myopia severity (**E**). REST-ANOVA analysis was performed to compare expression for each subgroup with respect to emmetropes used as a control (herein referred as 2, white box). Red dot lines are referred to 2log-FC PCR biological significance. *p*-values are shown by asterisks (* *p* ≤ 0.05; ** *p* ≤ 0.005; *** *p* ≤ 0.0005 and **** *p* ≤ 0.0001), as calculated by REST-ANOVA coupled to a Kruskal–Wallis post-hoc test.

RNA extracts were obtained from LEC and amplified to clarify the signaling of both factors. In Figure 2, the molecular data highlight an upregulation of *βNGF* transcripts (*p* < 0.005; Figure 2A) and a downregulation of *BDNF* (*p* < 0.005; Figure 2B) transcripts depending on myopia severity. Amplifications were also carried out for *NGF* and *BDNF*-specific receptors’ transcripts, showing a significantly low expression of *trkA^NGFR^* (Figure 2C) transcripts in high myopia with respect to myopia (*p* < 0.05) and emmetropia (*p* < 0.0005), and a significant deregulation of *trkB^BDNFR^* transcripts in myopia with respect to emmetrope (*p* < 0.0005; Figure 2D). Of interest, the pan-neurotrophin *p75^NTR^* receptor transcript expression was significantly upregulated in high myopia with respect to emmetrope (*p* < 0.0005; Figure 2E).

### 2.3. βNGF and BDNF Correlate with αSMA Expression (EMT Phenotype)

**Figure 3:**
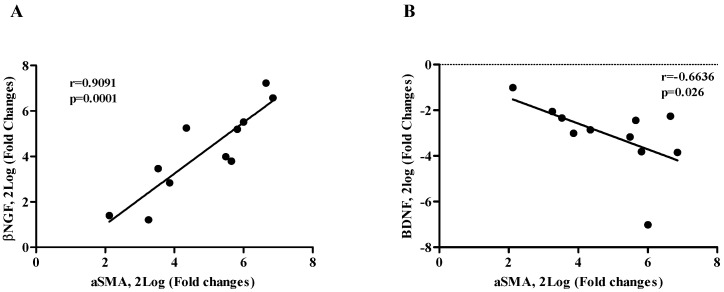
*βNGF* and *BDNF* correlations with *αSMA* in LEC. Scatterplots representative of *βNGF* (**A**) and *BDNF* (**B**) transcripts plotted against *αSMA*. The Spearman rho test showed that βNGF correlated positively with the contractile *αSMA* transcript expression (**A**). On the contrary, *BDNF* correlated negatively with respect to *αSMA* expression (**B**). Correlation data (coefficient and *p* value) are shown in the plot as 2log-expression values (fold changes).

Correlation analyses were carried out between αSMA and, respectively, *βNGF* and *BDNF* transcripts quantified in myopic LEC (Figure 3). The Spearman rho test analysis showed a direct correlation for *αSMA*/*βNGF* (r = 0.9091, *p* = 0.0001; Figure 3A), revealing an involvement of *βNGF* in the EMT process. A significant inverse correlation was noted for *αSMA*/*BDNF* transcripts (r = −0.6636, *p* = 0.026; Figure 3B).

### 2.4. NO and Nitrites Are Released in Myopic Aqueous and Correlate with NGF and BDNF

**Figure 4:**
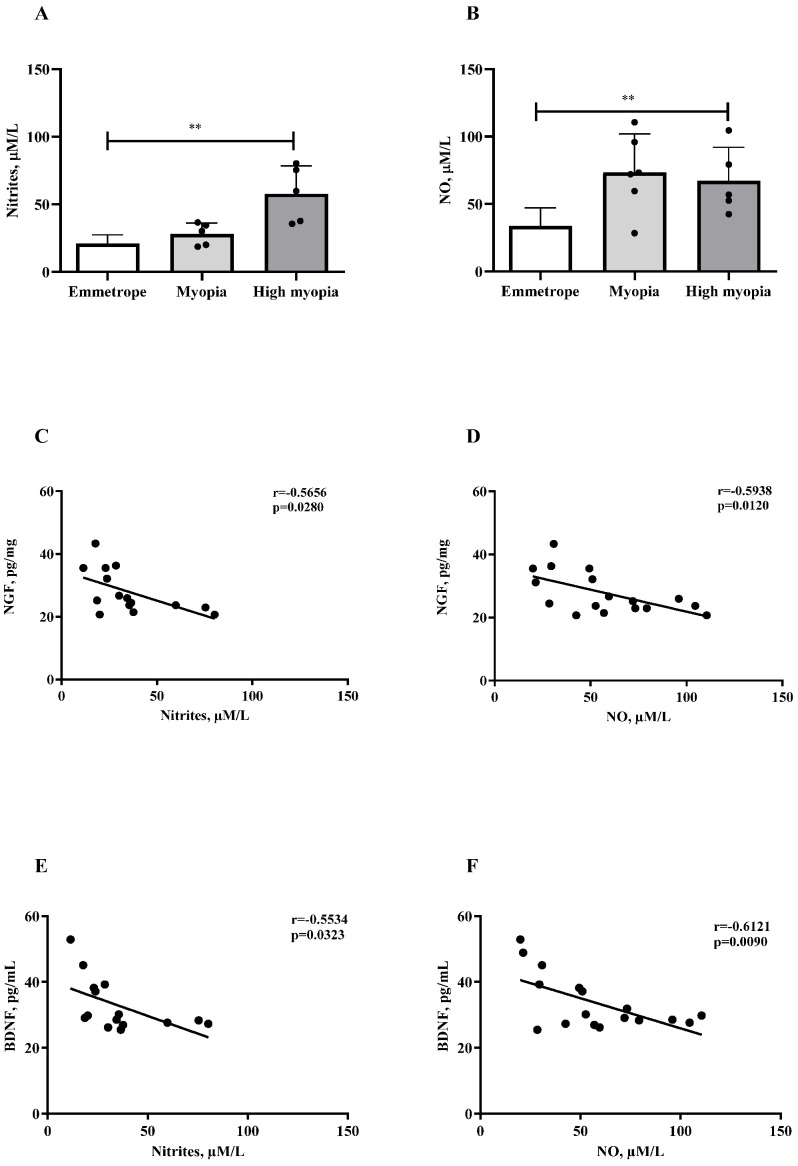
NO and nitrite correlations with NGF and BDNF. The bar graph shows nitrites (**A**) and NO (**B**) protein levels in humor aqueous from myopia and high myopia compared with emmetropia; the Pearson rho test showed inverted correlation between NGF/nitrites, NGF/NO, BDNF/nitrites, and BDNF/NO, respectively, in panel (**C**–**F**). Significant levels are shown in the panels (** *p* ≤ 0.005), as calculated using a Kruskal–Wallis test followed by a Dunn’s post-hoc test (mean ± SEM). Correlation data (coefficient and *p* value) are shown in the plot.

The oxidative stress parameter of nitrites and total nitric oxide in humor aqueous collected from myopic patients is shown in Figure 4A,B. The high myopia group levels significantly increased in respect to the myopia and emmetrope groups (*p* < 0.005). The scatterplots reveal NGF protein values negatively correlated with nitrites (r = −0.5656, *p* = 0.0280; Figure 4C) and NO (r = −0.5938, *p* = 0.0120; Figure 4D) protein values. A significant negative correlation was detected between BDNF/nitrites (r = −0.5534, *p* = 0.0323; Figure 4E) and BDNF/NO (r = −0.6121, *p* = 0.0090; Figure 4F).

### 2.5. Oxidative Stress Transcripts Expression in Anterior Capsule (LEC)

**Figure 5:**
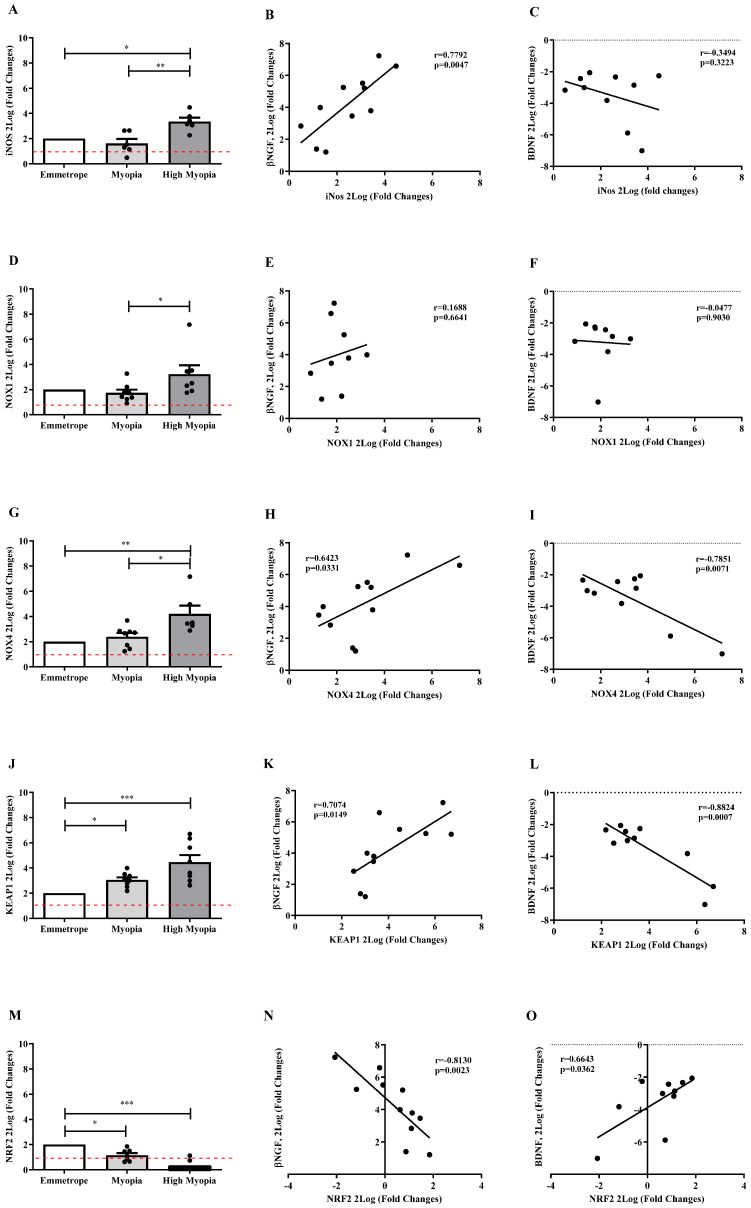
LEC and oxidative stress. Differences between high myopia and myopia are shown for *iNOS* (**A**) and related *NGF* and *BDNF* correlations (**B**,**C**), *NOX1* (**D**) and related *NGF* and *BDNF* correlations (**E**,**F**), *NOX4* (**G**) and related *NGF* and *BDNF* correlations (**H**,**I**), *KEAP1* (**J**) and related *NGF* and *BDNF* correlations (**K**,**L**), and *NRF2* (**M**) and related *NGF* and *BDNF* correlations (**N**,**O**) as indicated by asterisks according to the Kruskal–Wallis test followed by a Dunn’s post-hoc test (mean ± SEM) and correlation *p* values. Note the significant increase in *iNOS* mRNA (**A**), *NOX1* mRNA (**D**), *NOX4* mRNA (**G**), *KEAP1* mRNA (**J**), and the significant decrease in *NRF2* mRNAs (**M**) in high myopia vs. emmetropia. Note also the significant increase in *iNOS* mRNA (**A**), *NOX1* mRNA (**D**), and *NOX4* mRNA (**G**) in high myopia vs. myopia. *KEAP1* mRNA (**J**) and *NRF2* mRNA (**M**) were also significantly increased in myopia vs. emmetropia. Data are 2Log (fold changes, ±SEM), calculated with respect to emmetropic eyes used as controls and referred to as 1 (white box). Red dotted lines indicate the level of significance for relative PCR. Significant levels are shown as calculated using one-way ANOVA analysis (* *p* ≤ 0.05; ** *p* ≤ 0.005; and *** *p* ≤ 0.0005). Correlation data (coefficient and *p* value) are shown in the scatterplots’ representation of *βNGF* and *BDNF* transcripts (2Log, fold changes) plotted against oxidative stress mediators (2Log, fold changes).

Regarding the oxidative stress-related enzymes, a significantly high expression of the inducible *iNOS* (Figure 5A) as well as the nitric oxide enzymes *NOX1* (Figure 5D) and NOX4 (Figure 5G) transcripts was observed, specifically in high myopia, with respect to emmetropia (respectively, *p* < 0.05, *p* ≥ 0.05 and *p* < 0.005), and between myopic subgroups (*p* < 0.005, *p* < 0.05, and *p* < 0.05). More interestingly, *KEAP1*mRNA expression was upregulated (emmetrope vs. high myopia: *p* < 0.0005, emmetrope vs. myopia: *p* < 0.05; Figure 5J), while *NRF2*mRNA expression was deregulated (emmetrope vs. high myopia: *p* < 0.0005, emmetrope vs. myopia: *p* < 0.05, Figure 5M) depending on disease severity. The Pearson rho test analysis showed that *βNGF* correlated positively and significantly with *iNOS* (r = 0.7792, *p* = 0.0047; Figure 5B), *NOX4* (r = 0.6423, *p* = 0.0331; Figure 5H), and *KEAP1* (r = 0.7074, *p* = 0.0149; Figure 5K), but negatively with *NRF2* (r = −0.8130, *p* = 0.0023; Figure 5N) transcript expression. On the contrary, *BDNF* correlated negatively and significantly with respect to *NOX4* (r = −0.7851, *p* = 0.0071; Figure 5I) and *KEAP1* (r = −0.8824, *p* = 0.0007; Figure 5L), but positively with respect to *NRF2* (r = 0.6643, *p* = 0.0362; Figure 5O) expression. Insignificant data correlation was observed between *βNGF*/*NOX1* (Figure 5E), *BDNF*/*NOX1* (Figure 5F), and *BDNF*/*iNOS* (Figure 5C).

### 2.6. Epigenetic Mediators Are Activated in Anterior Capsule Myopic LEC

**Figure 6:**
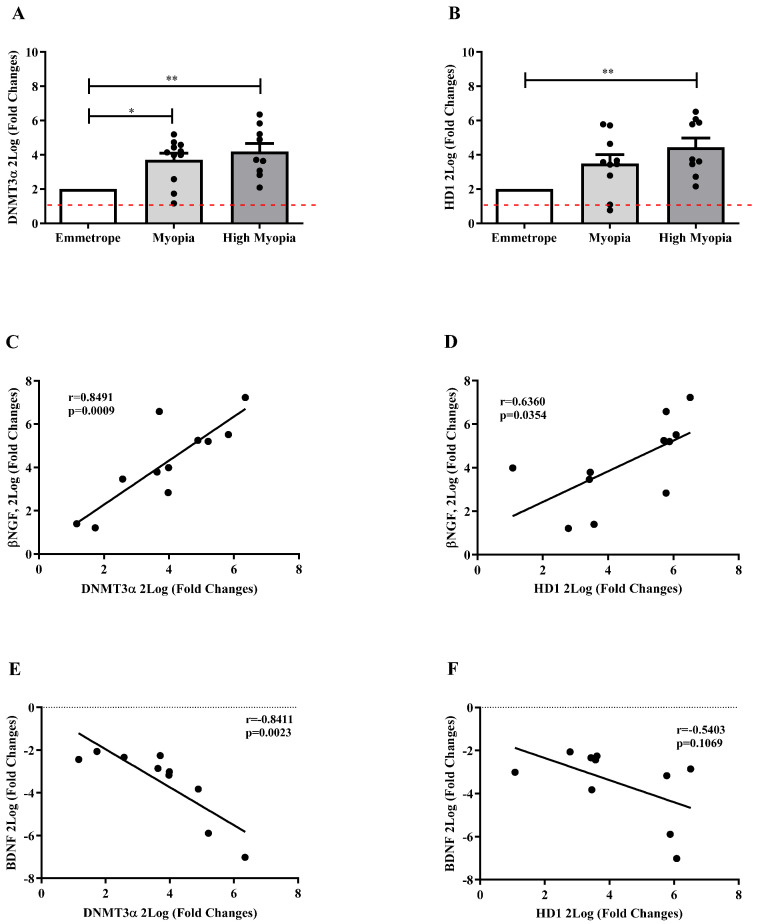
LEC and epigenetic mediators. Bar graph showed *DNMT3α*mRNA (**A**) and *HD1*mRNA (**B**) increased expression depending on myopia severity in LEC. Particularly, significant changes were observed for *DNMT3α*mRNA (**A**) and *HD1*mRNA (**B**) between high myopia and emmetropia, and for *DNMT3α*mRNA (**A**) between myopia and emmetropia. No significant changes were observed for both *DNMT3α*mRNA and *HD1*mRNA (**B**) between high myopia and myopia (**A**,**B**). Significant levels are shown in the panels (* *p* ≤ 0.05; and ** *p* ≤ 0.005), as calculated using the Kruskal–Wallis test followed by a Dunn’s post-hoc test (mean ± SEM). Red dot lines are referred to 2log-FC PCR biological significance. The Pearson rho test showed direct correlation between *βNGF*/*DNMT3α* (**C**) and *βNGF*/*HD1* (**D**) and an inverted correlation between *BDNF*/*DNMT3α* (**E**) and *BDNF*/*HD1* (**F**). Correlation data (coefficient and *p* value) are shown in the plot.

Epigenetic mediators were herein investigated and correlated with *βNGF* and *BDNF* signaling. As shown, *DNMT3α* (Figure 6A) and *HD1* (Figure 6B) mRNA levels were significantly upregulated in LEC collected from patients with myopia for *DNMT3α* (*p* < 0.05) and high myopia (respectively, *p* < 0.005 and *p* < 0.005), as compared to emmetrope. Both epigenetic mediators showed a significantly direct correlation with *βNGF* (r = 0.8491, *p* = 0.0009; Figure 6C and r = 0.6360, *p* = 0.0354; Figure 6D) and an inverted significant correlation between *BDNF*/*DNMT3α* (r = −0.841, *p* = 0.0023; Figure 6E), but not significant between *BDNF*/*HD1* (Figure 6F).

### 2.7. Anterior Capsule Myopic LEC Differentially Express iNOS and cJun

**Figure 7:**
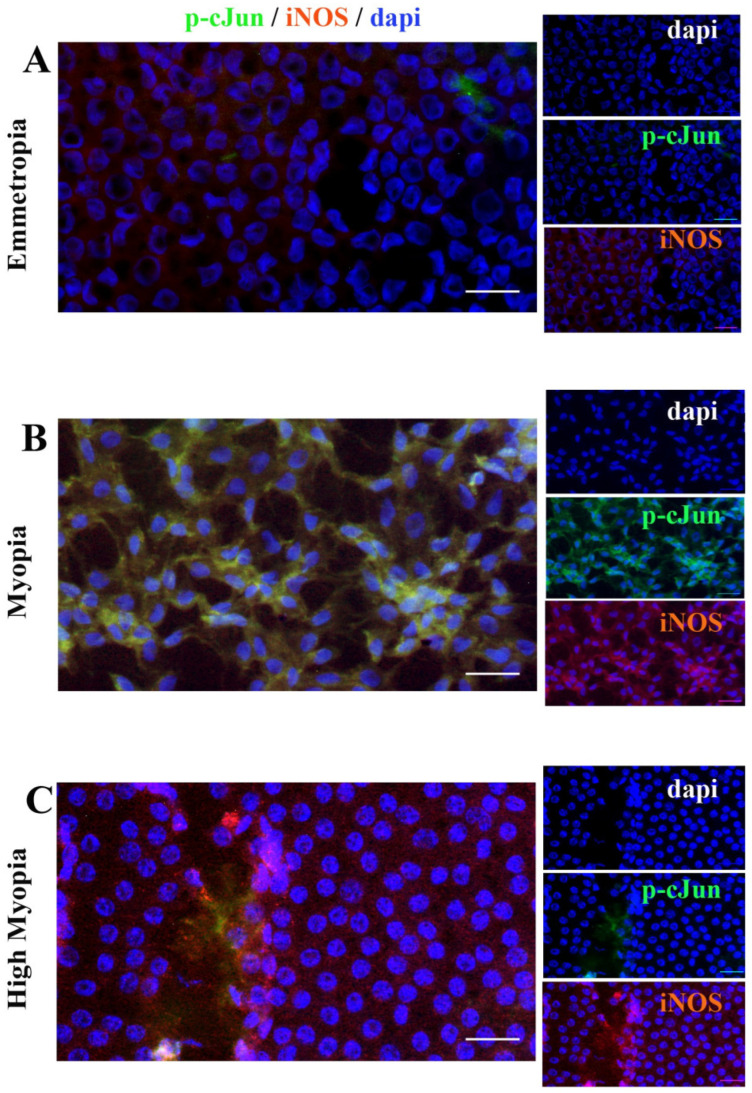
cJun and iNOS protein expression in LEC. Representative merged acquisitions for cJun (green) and iNOS (red) at emmetropia (**A**), myopia (**B**), and high myopia (**C**). LEC monolayers were counterstained with nuclear DAPI (blue) to better visualize the monolayers with a regular distribution. Note the significant increase in cJun immunoreactivity in myopic LEC (**B**) and iNOS immunoreactivity in high-myopic LEC (**C**). Data represent mean ± SEM, and values of fluorescent intensity (IntDen; ImageJ version 1.54p) are expressed in arbitrary units. Magnifications ×400 (white bar size = 50 μm).

Since trkA^NGFR^ and p75^NTR^ transcripts were overexpressed in myopic and high-myopic LEC, cJun and iNOS immunoreactivity were explored in few representative monolayers. Briefly, both cJun (green/cy2) and iNOS (red/cy3) were probed in emmetropia, myopic and high-miopic LEC’ monolayers, and analyzed by epifluorescent analysis (×400). Particularly, the highest cJun expression was observed in myopic LEC (Figure 7C) as compared to high-myopic and emmetropic LEC (respectively, Figure 7B and Figure 7A). Immunoreactivity specific to iNOS was observed in all subgroups, increasing at myopic (Figure 7A) and further at high-myopic (Figure 7A) LEC. This increased trend of immunoreactivity corroborates the molecular data.

## 3. Discussion

**Figure 8:**
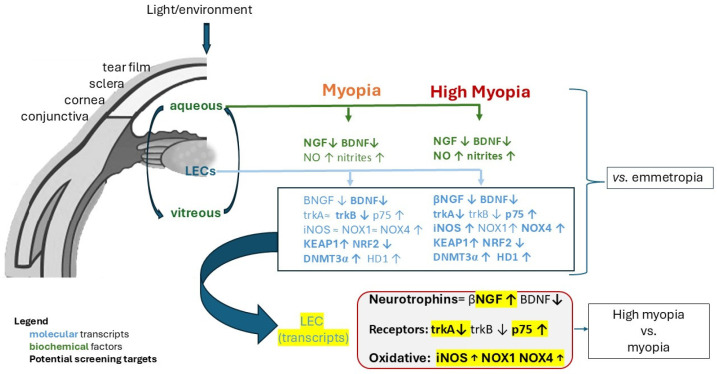
Schematic representation of the main outcomes of the study. Graphical abstract reporting the molecular, biochemical, and potential screening (high myopia vs. myopia) targets over an open schematic eyeball. NGF, BDNF, oxidative stress and epigenetic results are shown in bold if significantly expressed in both aqueous (protein) and LEC (transcripts) extracts from myopia and high myopia. The differential pattern of expression between NGF and BDNF, both proteins and transcripts, would suggest the involvement of both neurotrophins at any stage of myopia, as suggested by the upregulation of the pan-neurotrophin *p75^NTR^* receptor. Oxidative stress (*iNOS* and *NOX4*) targets are overexpressed at high myopia, while a high *KEAP1*/*NRF2* ratio was observed in both cases. Finally, an epigenetic involvement was observed by the upregulation of both *DNMT3α* and *HD1* enzymes. Form the analysis of the targets (see black frame), a list of potential candidate «screening» targets between myopia and high myopia (red frame) appear as suitable differential profile targets of expression (yellow).

Herein, we report the differential expression of NGF, BDNF, and related receptors between myopia and high myopia, and their association with NO/nitrites/iNOS/NOX1/4, KEAP1/NRF2, and some epigenetic factors (HDMT3α, HD1), as observed from studies on aqueous and myopic LEC. A schematic illustration of the results of this study is shown in Figure 8.

Myopia is a refractive error leading to blurred vision of images from a distance, the most prevalent causes of visual impairment, and high myopia is unfortunately associated with vitreo-retinal interface anomalies and retinal degeneration, as the main complication is represented by myopic CNV (mCNV) [1,2,3,4]. In a recent study, we described the differential protein and transcript expression profile of inflammatory mediators between myopia and high myopia [14]. Merely, we investigated whether a high-myopic aqueous profile might be rich in potential candidates for rating anterior chamber inflammation and even predicting retinal distress if collected at the time of cataract surgery [14]. Particularly, we observed that a strong link occurs between AH protein signature and EMT-LEC phenotype [14]. Furthermore, the low VEGF-A/ANG-2 and the high VEGF-A/VEGF-D ratios in myopic AH might suggest a specific inflammatory and profibrogenic pattern in high myopia [14]. Some proteomic studies revealed that proteins associated with the complement system, ECM remodeling, and mitochondrial energy metabolism might be key effectors in myopiagenesis [26]. Since not all high-myopic eyes develop mCNV, implying the existence of some neuroprotective mechanisms, we hypothesized the participation of NGF and BDNF as well as few oxidative-related and epigenetic factors in the progression of this refractive error [17,18,19,20,32].

First, we observed a significant decrease in NGF and BDNF protein levels in aqueous from myopic subjects, with no difference between myopic subgroups. The decreased NGF content in aqueous was associated with an increased NGF transcript expression by LEC, while the decreased BDNF content was in line with the decreased BDNF transcript expression by LEC. The presence of NGF and BDNF in myopic aqueous might be due to the local production from the ciliary process, corneal endothelial cells, and LEC, particularly if undergoing an EMT conversion [10,14]. The observation of an increase in NGF transcripts by LEC might have at least two possible explanations: the first is related to the NGF autocrine utilization by LEC under EMT changes, or a paracrine utilization by surrounding LEC and other cell types belonging to the anterior chamber (such as corneal endothelial cells and tendon fibroblasts), and second is the necessity of a general neuroprotection against oxidative stress or neuroprotection [14,17,18,19,20]. Instead, BDNF outcomes seem to support the hypothesis that this neurotrophin is not involved in the EMT-LEC process nor in the local inflammation [33]. In previous studies, NGF and BDNF applications to the eye provided great value for tear film stabilization, ocular surface and retinal neuroprotection, as well as ganglion cell survival [19,20]. The fact that only βNGF correlated positively with smooth muscle actin (αSMA) expressed by LEC, while BDNF and αSMA were negatively related, would suggest that only NGF could be involved in the transepithelial differentiation (EMT). This aspect is in line with previous studies on cells belonging to the anterior segment, and NGF was reported as a profibrogenic factor associated with EMT properties [17,33]. The correlation studies expanded our previous studies on the transforming growth factor (TGF)β1 and receptor-mediated EMT differentiation of myopic LEC [14].

Both NGF and BDNF signal by two specific receptors and one pan-neurotrophin glycoprotein [17,18]. Herein, we observed the downregulation of trkA^NGFR^ transcripts and the upregulation of p75^NTR^ transcripts in high-myopic LEC. This finding is extremely important, as it would suggest that a possible regulatory effect is played by NGF or proNGF. This decreased trkA^NGFR^/p75^NTR^ ratio in high-myopic LEC reinforces the contribution of NGF with respect to BDNF [34].

Oxidative stress is one of the main mechanisms occurring in LEC affected by high-myopic cataract and is most probably associated with an imbalance of mitochondrial homeostasis, as previously reported [35]. Since the age-related cataract is closely associated with oxidation, calcium imbalance, impairments in cytokine/chemokine profiles, hydration, and crystallin modifications, we bypassed this mediator bias by comparing all subgroups coming from similar cataract evolution [23]. Well-known mediators of oxidative stress were investigated and compared in myopic and high-myopic LEC. Nitrite is a physiological reservoir of NO and a modulator of mitochondrial function [25,26]. Mitochondrial oxidative stress has been demonstrated in retinal and macular diseases and recently it has been implicated in myopia and high myopia development and progression [35,36]. Herein, the purpose of measuring the levels of nitrites and NO was twofold: on one hand, nitrites are indicators of NO production and nitrites are the main markers of oxidative stress [35,36]. Regarding the increased levels of nitrites and NO observed in myopic aqueous, it is important to highlight that both mediators play a role in the development and progression of myopia, potentially through their direct influence on the ciliary muscle [37]. These findings are of interest as the ciliary muscle contraction increases the refractive power (accommodation) of crystalline lens, representing one of the risk factors for myopia progression [38]. To support this, different experimental studies are available on NO as modulators of high myopia [39]. Nitrites and NO transcripts’ expression in LEC also correlated with NGF and BDNF transcript expression in LEC. Particularly, the levels of nitrites and NO correlated negatively with NGF and BDNF. These findings are in line with previous studies on the NO and NGF relation in several diseases, which is in line with the protective antioxidant properties of NGF [40].

Oxidative stress is frequently associated with the inducible form of NOS (iNOS) and the NOX family (NADPH oxidase), comprising three soluble isoforms (NOX1, NOX2, and NOX4) [40,41]. Herein, we observed an increased expression of transcripts specific to iNOS and two metabolic enzymes, NOX-1 and NOX-4, and these targets were particularly increased in high myopia with respect to myopia. Data from the literature indicate that iNOS is often associated with NGF and BDNF expression due to the relation to NO that is also a neurotransmitter [39]. Regarding the NADPH oxidase, NOX1 is the major mediator of oxidative-derived ROS production (O^2−^), and its local activation triggers the excessive production of superoxide, leading to cell apoptosis, and NOX4 is the only family member that generates hydrogen peroxide (H_2_O_2_) [42,43]. iNOS, NOX1, and NOX4 transcripts’ expressions are significantly different between myopia and high myopia, implying a differential oxidative stress signature.

Furthermore, the imbalance between oxidative stress, NGF, and BDNF in human high myopia, which showed an association with total nitrite levels, was also observed. The total nitrate parameter has been traditionally used as an oxidative stress marker, suggesting that NO, specifically through peroxynitrite, could be considered one of the sources of oxidative stress [40]. NGF-induced ROS is not a deleterious event per se, in line with the concept that both NGF and NO might act as signaling molecules during differentiation [41]. Vice versa, ROS-induced NGF might have potential implications in tissue neuroprotection [41,42,43].

The KEAP1/NRF2 signaling pathway is a powerful cellular defense system against oxidative damage [44,45]. Our studies showed that keap1 transcripts were high in high myopia, with no difference between myopia subgroups, and nrf4 transcripts were low in both myopia and high myopia. This finding can be explained as the overexpressed KEAP1 significantly decreasing the levels of NRF2, and the lower levels of NRF2 inducing loss of the redox balance toward oxidative stress, leading to failure of lens cytoprotection [44,45]. As an important nuclear transcription factor, NRF2 modulates the transcription of various antioxidant genes by binding to antioxidant response elements (AREs) in DNA promoters, thereby maintaining redox homeostasis and driving the upregulation of cytoprotective enzymes, attenuating the cataract process [45]. Total and nuclear NRF2 expressions in lenses were dramatically reduced in age-related and diabetic cataracts, suggesting that NRF2 can play an antioxidant role during aging [27].

As above introduced, myopic LEC appears to be under epigenetic control. Since DNA methylation is mediated by DNMT, while DNA acetylation is carried out by HD1, their transcript expression was also investigated in myopic and high-myopic LEC [46,47]. In our study, DNMT3α and HD1 transcripts were both upregulated in high myopia, and particularly these expressions were positively related to NGF and negatively related to BDNF. This aspect is not new as cataract and myopia show common light exposure, and NGF was found to protect corneal cells and retina from the UV light effects, both in vitro and in experimental models [48]. A good question might be clarifying the specific regulations of NGF and BDNF transcription in high myopia, and the possibility of using DNMT inhibitors that can reverse the hypermethylation status of these mediators if detected.

Finally, the observation of an increased immunoreactivity specific to iNOS in high-myopic LEC and phospho-cJun in myopic LEC would reinforce the contribution of p75^NTR^ as discriminating receptors in these two forms of myopia.

In the last ten years, researchers gained information on NGF and BDNF immune-modulatory effects at the ocular surface and retina [14,19,20,49,50]. Some studies focused on the role of TGF-β in myopia development, being a key regulator of scleral remodeling, which can contribute to eye elongation and myopia progression, prospecting the hypothesis that NGF and BDNF might also act as additional remodeling factors [6,51,52,53]. Although NGF and other growth factors can promote nerve regeneration, increase corneal sensitivity, and improve tear film stability, their involvement in the remodeling of the refractive apparatus appears of interest due to its ECM and collagen remodeling properties [17,33]. With respect to pathological myopia, the prolonged wear of long-term soft contact lenses in subjects with high myopia was associated with increased levels of corneal stromal neuromediators and reduced corneal optical quality [20,54]. The hypothesis of the use in the near future of the recently tested engineered scaffolds able to release rhNGF in vivo could appear of interest not only for neurodegenerative disorders [55].

Taken together, changes in NGF, BDNF, and NO levels in blood and ocular fluids have been reported during the onset and progression of many illnesses, including ophthalmological, neurological, psychiatric, endocrine, and immune diseases and other physio-pathological disorders, such as cardiometabolic states, stressful events, alcoholism, and aging [56]. Herein, we prospect the contribution of NGF and BDNF, throughout transcript and receptors modulation, in myopia and high myopia, prospecting their involvement at both inflammatory and neuroprotective levels.

## 4. Materials and Methods

This observational study was conducted in accordance with the ethical standards stated in the Declaration of Helsinki and was approved by the intramural ethical committee (IFO/Bietti, Rome, Italy, number 99/20/FB). Patients provided written adherence to the protocol by signing the informed consent before their enrollment in the study and the clinical and biostrumental ophthalmological examination and finally the collection of biosamples during cataract surgery.

### 4.1. Study Population: Clinical Assessment and Sampling

Sixty-five eyes (65; 29M/36F) from patients with cataracts were enrolled in the study (June 2019–June 2021). Eyes were grouped based on both axial length (AL) and refractive error (RE, expressed as spherical equivalent). Emmetropia was defined as SE between −0.50 D and +0.50 D; myopia as SE ≤ −0.50 D and AL between 24 and 26 mm; and high myopia as SE ≤ −6.00 D and AL ≥ 26 mm. Biosamples were grouped as emmetrope (23; 81.00 ± 6.70 years); myopia (24; 75.96 ± 7.30); and high (pathological) myopia (18; 70.56 ± 11.68 years), depending on axial length (AL) and refractive error (RE).

These biostrumental data were collected at the time of complete ophthalmological visit for study recruitment. The recruitment was carried out according to inclusion (primary myopia) and exclusion criteria (previous intraocular surgery, other ocular diseases, presence of hyper-mature cataracts, anti-VEGF intravitreal injections, and topical anti-glaucoma therapy). Patients with systemic neurodegenerative diseases (Alzheimer’s or Parkinson’s diseases) or local/systemic autoimmune diseases (merely Sjogren’s syndrome and diabetes), endocrine (thyroiditis), and metabolic (diabetes) diseases were also excluded from the study. Patients underwent pre-surgical ophthalmological examination, including anamnesis, comorbidities, optometric tests (AL measurement), and slit-lamp assessment with complete pupillary dilation.

### 4.2. Pre-Analytical Procedures

Aqueous humor samples (10–200 µL) were sampled at the beginning of surgery and immediately after incision by a sterile syringe, and anterior-capsule-bearing lens epithelial cell (AC/LEC) specimens were sampled during capsulorhexis. AH samples were quickly centrifuged (1300 rpm/7 min), and cell-free supernatants were collected and supplemented with protease inhibitors (Pierce; Thermo Fisher Scientific, Milan, Italy). Samples were spectrophotometrically analyzed (Nanodrop N1000; Celbio, Euroclone S.p.A, Milano, Italy) and stored at −20 °C. AC specimens were devoted to LEC analysis according to a standard procedure.

### 4.3. Biochemical Analysis

Forty-four (44) AH samples were analyzed for protein assays (ELISA). Conventional DuoSet^®^ Kits were used for human βNGF (DY256-05) and BDNF (DY248-05) detection (R&D Systems, McKinley Place, Minneapolis, MN, USA). Briefly, 96-well MaxiSorp™ plates (Nunc, Roskilde, Denmark) were pre-coated with the specific capture antibodies (0.4 μg/mL; 4 °C/overnight; R&D). Samples were appropriately diluted (1:2 for NGF and 1:3 for BDNF) in assay diluent (R&D) supplemented with 1× protease inhibitor cocktail (Pierce-Thermo Fisher Scientific Inc., Waltham, MA, USA) and loaded in precoated plates in parallel with the standard curve (0.32–2.000 pg/mL protein; R&D). Subsequently, the specific secondary antibodies (0.15 μg/mL; R&D) and streptavidin (1:200; R&D) were added, and the specific binding was developed by using the ready-to-use 3,3′,5,5′-Tetramethylbenzidine (TMB) substrate solution (R&D). The colorimetric signals (optic density, OD) were acquired at λ 450–570 nm by using a 96-well plate reader (spectrophotometer; Tecan Group Ltd., Männedorf, Switzerland). Target values were normalized for total protein (A280; Nanodrop analysis) and were produced using a 3rd grade polynomial standard curve, as calculated by Prism 9.3 (GraphPad Software Inc., San Diego, CA, USA). Concentrations of analytes are shown as pg/mL. The absence of cross reactivity with other neurotrophins was assured by the manufacturers. The analysis was performed by an operator without knowledge of the clinical information of the sample handled. The total Nitric Oxide Assay Kit (EMSNOTOT, ThermoFisher Scientific, Waltham, MA, USA) was used to quantify all-in-one both nitrites and nitric oxide concentrations based on the enzymatic transformation of nitrate into nitrite by nitrate reductase. The reaction was carried out according to the manufacturer’s instructions and the colorimetric detection mode of nitrite was used as an azo dye product of the Griess reaction. Concentrations of analytes are shown as µM/L.

### 4.4. Molecular Analysis: Two-Step Real Time PCR

Fifty-one (51) AC/LEC samples were used for molecular evaluations. Total RNAs were extracted according to the TRIfast technique (Euroclone); treated with DNAseI (AB1709; Ambion Inc., Austin, TX, USA); and spectrophotometrically analyzed for 260/280 ratio and RNA quantity (Nanodrop N1000 Spectrophotometer; Celbio Euroclone). RNA quality was verified randomly in 1% agarose gel electrophoresis (Promega, Madison, WI, USA) in a horizontal minicell chamber (Biorad, Hercules, CA, USA). Only samples with a 260/280 ratio > 1.8 were used for cDNA synthesis, starting from 3 µg total RNA and using the GoScript standardized procedure (Promega, Madison, WI, USA), including random hexamers and dNTs (Promega, Madison, WI, USA) in a OneCycle programmable thermocycler (LifePRO/BIOER; Euroclone, Milan, Italy). For amplifications, 3 µL cDNAs for the target gene (1 µL for reference ones) was amplified in a 20 µL final volume of SYBR Green PCR mixture (Hot-start SYBRgreen Hydra mix) using the Biorad CFX96 Real-Time PCR System (BioRad, Hercules, CA, USA). Amplification profile was as follows: one cycle of 95 °C/5 min initial denaturation followed by 35–40 cycles at 95 °C/30 s (denaturation), 58–61 °C/25 s (annealing temperature; AT), and 72 °C/30 s (elongation), followed by fluorescence monitoring at 60–90 °C, 0.01 °C for 0.3 s, and further elongation at 72 °C/5 min. Negative and positive controls were run in parallel according to a standard procedure. The specific pair primers and the accession number of genes are shown in Table 2.

### 4.5. Immunofluorescence and Epifluorescent Microscopy

A total of 6 anterior capsules bearing intact LEC (2 for emmetrope; 2 for myopia; and 2 for high myopia) were used for immunofluorescent analysis and epifluorescent aquisition. Briefly, citofixed LECs placed on an intact anterior capsule were briefly rehydrated in PBS (10 mM phosphate buffer (PB) and 137 mM NaCl; pH 7.5). A blocking/permeabilizing step (0.1% BSA and 0.3% Triton X100 in PBS) was carried out before the specific antibody incubation. Probes were the polyclonal anti-human phospho-cJun antibody (Goat; 1:200; Santa Cruz, CA, USA) and polyclonal anti-human iNOS antibody (rabbit; 1:200; Santa Cruz Biotech., Santa Cruz, CA, USA). Specific bindings were detected using secondary AlexaFluor-4888 or AlexaFluor-555-coupled anti-goat (for p-cJun) or anti-rabbit (for iNOS) species-specific F(ab)2 antibodies diluted (1:500) in 0.05% Tween20-PBS and incubated for 45 min on a benchtop (Immunological Sciences, Rome, Italy). After nuclear counterstaining (blue/DAPI; Molecular Probes, Eugene, OR, USA), the specimens were mounted with an anti-fading supplemented Vectashield (Vector Laboratories, Inc., Burlingame, CA, USA) and examined under an epifluorescent direct microscope (Ni-Eclipse; Nikon, Tokyo, Japan) equipped with a UV lamp and digital camera (Axiocam; Carl Zeiss, Jena, Germany). The free available ZEN 3.1 acquisition software (blue edition; Zeiss) was used for acquisitions (×40) and format refining (8-tiff format) for both single and merged images.

### 4.6. Statistical Analysis

A formal sample size calculation was performed at the beginning of the study with the preset parameters: effect size d = 0.5 (medium); error probability = 0.05; and an allocation ratio of 1 (Genetic Power Calculator G Power 3.1.9.4; free available) considering two populations and the main two biomarkers (NGF and BDNF). The real power of this study population, for a total size of 65 subjects, considering means from these two independent groups (42 case and 23 controls) for NGF and BDNF, was 0.776. Values were analyzed using the Shapiro–Wilk test and F test to satisfy normality and variance assumptions. Graphical representations, descriptive statistics, Pearson test, and Kruskal–Wallis coupled with Dunn’s post-hoc analyses were carried out for identifying significant differences between groups (Prism vs. 10.0.0; GraphPad Software Inc., San Diego, CA, USA). For molecular analysis, only normalized samples (run in duplicate) were amplified, and cycle threshold (Ct) values from good melting curves were used for analysis in the REST© software ver. 2. Fold changes were calculated as the expression level of the target transcript with respect to the reference one (H3), considering treated versus untreated ones (2log scale). According to the REST formula, values inside the [−2 and +2] range were not considered to have biological relevance, and a red dashed line was introduced in the graphics. The Pearson test was used to correlate protein data, protein data with AL elongation, and transcripts data. Significant levels are shown in the panels (ns *p* ≥ 0.05; * *p* ≤ 0.05; ** *p* ≤ 0.005; *** *p* ≤ 0.0005; and **** *p* ≤ 0.0001), as calculated using Kruskal–Wallis followed by a Dunn’s post-hoc test (mean ± SEM).

## 5. Conclusions

Myopia is a world-wide health concern affecting childhood and adulthood in a percentage of 22.9% of the entire population, and it has been estimated that this percentage will increase to 49% in 2050 [1,2,3]. Overall, inflammation is a protective mechanism used by multi-cellular eukaryotic organisms to remove the insults and trigger the healing process. In myopia, progressive visual axial elongation is a process characterized by long-lasting tissue remodeling, and it is associated with inflammation [51,52,53,54,55,56]. To date, high myopia is thought to be an inflammation-related disease as suggested by the findings on myopia-mediated retinal complications such as mCNV characterized by inflammation and vascularization [14,29,30,31]. An interesting aspect of myopia progression is the accumulation of oxidative damage in the lens, leading to crystallin aggregation and apoptosis of LEC [32]. Herein, our findings on differential expression/regulation of NGF and BDNF and the association with oxidative and epigenetic targets would imply the possibility to consider the neuroinflammation and the neuroprotection, mainly via NGF and p75^NTR^, as an active part of disease progression.

## Figures and Tables

**Table 1:** Study population.

	Population	Emmetropia	Myopia	High Myopia	*p* Value
Patients	65	23	24	18	
Mean age ± SD	76.25 ± 9.40	81.00 ± 6.70	75.96 ± 7.30	70.56 ± 11.68	*p* > 0.05
Sex F/M	36/29	12/11	12/12	12/6	*p* > 0.05
Axial length	25.52 ± 2.74	23.14 ± 0.45	24.83 ± 0.50	29.46 ± 1.68	*p* < 0.05

Clinical and biostrumental examinations were performed at the time of recruitment, and patients were classified according to axial length (AL) elongation values (emmetropia, myopia, and high myopia). Anamnesis included family history and systemic and local comorbidities. Data are mean ± SD. Statistically significant *p* values were obtained according to the one-way ANOVA followed by a Tukey’s HSD post-hoc test. *p* value ≤ 0.05 indicates that the difference is statistically significant, as shown in bold. M, male; F, female; and age (years).

**Table 2:** Primer sequences and accession number of amplicons for relative real-time PCR.

Genes	Primer Sequence		GeneBank
Reference gene			
*18S*	5′-GGAGAGGGAGCCTGAGAAAC-3′	5′-AGGGCCTCGAAAGAGTCCT–3′	M10098
*H3*	5′-GTCTGCAGGCTGGCATAGAAG-3′	5′-TCGCCTTCTGGGTTGAGTG-3′	NM005324.4
Target genes			
*βNGF*	5′-TGAAGCTGCAGACACTCAGG-3′	5′-CACCTCCTTGCCCTTGATGT-3′	BC126150.1
*BDNF*	5′-CCT TT GAG CCT CCT CTT CTC-3′	5′-ACT GTC ACA GCT CAG CTC-3′	M61176.1
*trkA*	5′-GCT CAG TCG CCT GAA TCT CT-3′	5′-GCA CAA GAA CAG TGC AGA GG-3′	M23102
*trkB*	5′-TGG TGG TGA TTG CGT CTG-3′	5′-CCA CA CCC CTT TCT CTG T-3′	AF400441
*p75*	5′-CCT ACG GCT ACT ACC AGG ATG AG-3′	5′-TGG CCT CGT CGG AAT ACG-3′	AF187064
*iNOS*	5′-CCCCTTCAATGGCTGGTACA-3′	5′-GTTTCCAGGCCCATTCTCCT-3′	U31511.1
*NOX1*	5′-CCAGGATTGAAGTGGATGGT-3′	5′-AGGTTGTGGTCTGCACACTG-3′	BC075014.2
*NOX4*	5′-CTCAGCGGAATCAATCAGCTGTG-3′	5′-AGAGGAACACGACAATCAGCCTTAG-3′	BC040105.1
*KEAP1*	5′-TTCAGCTACACCCTGGAGGA-3′	5′-CTTGAAGACAGGGCTGGATG-3′	BC002417.2
*NRF2*	5′-ACACGGTCCACAGCTCATC-3′	5′-TGCCTCCAAAGTATGTCAATCA-3′	BC011558.1
*DNMT3α*	5′-GCA CTC AAG GGC AGC AGA TA-3′	5′-TTC CAG GCT TCC AGG GTT AG-3′	C032392
*HD1*	5′-GGG ATC GGT TAG GTT GCT TC-3′	5′-AGG GCC ACA GCT GTC CTC ATA-3′	U50079

Specific amplifications were tested by verifying the single curve specific for each amplicon. Hot-start SYBRgreen Hydra mix was activated by a pre-hold (5 min at 50 °C) and pre-incubation for 15 min at 95 °C. Each of the 39 amplification cycles consisted of a 30 s/94 °C (denaturation), followed by a specific annealing step 58–60 °C and a 30 s/72 °C (extension). Annealing was set at the appropriate temperature (Tm—5 °C) verified for specificity by grading. The melting curve was registered from 56.0 °C to 94.1 °C; 0.3 °C; hold for 00:00:01 between reads.

## Data Availability

Data is contained within the article.
